# The White Matter Integrity and Functional Connection Differences of Fornix (Cres)/Stria Terminalis in Individuals with Mild Cognitive Impairment Induced by Occupational Aluminum Exposure

**DOI:** 10.1523/ENEURO.0128-24.2024

**Published:** 2024-08-20

**Authors:** Feifei Zhang, Yangyang Li, Ruihong Chen, Pengxin Shen, Xiaochun Wang, Huaxing Meng, Jiangfeng Du, Guoqiang Yang, Bo Liu, Qiao Niu, Hui Zhang, Yan Tan

**Affiliations:** ^1^Departments of Radiology, First Hospital of Shanxi Medical University, Taiyuan, Shanxi Province 030001, P.R. China; ^2^Shanxi Key Laboratory of Intelligent Imaging and Nanomedicine, First Hospital of Shanxi Medical University, Taiyuan, Shanxi Province 030001, P.R. China; ^3^Departments of College of Medical Imaging, Shanxi Medical University, Taiyuan, Shanxi Province 030001, P.R. China.; ^4^Occupational Health, School of Public Health, Shanxi Medical University, Taiyuan, Shanxi Province 030001, P.R. China.

**Keywords:** functional connectivity, mild cognitive impairment, occupational aluminum exposure, resting-state functional magnetic resonance imaging, white matter

## Abstract

Long-term aluminum (Al) exposure increases the risk of mild cognitive impairment (MCI). The aim of the present study was to investigate the neural mechanisms of Al-induced MCI. In our study, a total of 52 individuals with occupational Al exposure >10 years were enrolled and divided into two groups: MCI (Al-MCI) and healthy controls (Al-HC). Plasma Al concentrations and Montreal Cognitive Assessment (MoCA) score were collected for all participants. And diffusion tensor imaging and resting-state functional magnetic resonance imaging were used to examine changes of white matter (WM) and functional connectivity (FC). There was a negative correlation between MoCA score and plasma Al concentration. Compared with the Al-HC, fractional anisotropy value for the right fornix (cres)/stria terminalis (FX/ST) was higher in the Al-MCI. Furthermore, there was a difference in FC between participants with and without MCI under Al exposure. We defined the regions with differing FC as a “pathway,” specifically the connectivity from the right temporal pole to the right FX/ST, then to the right sagittal stratum, and further to the right anterior cingulate and paracingulate gyri and right inferior frontal gyrus, orbital part. In summary, we believe that the observed differences in WM integrity and FC in the right FX/ST between participants with and without MCI under long-term Al exposure may represent the neural mechanisms underlying MCI induced by Al exposure.

## Graphical abstract

**Figure:**
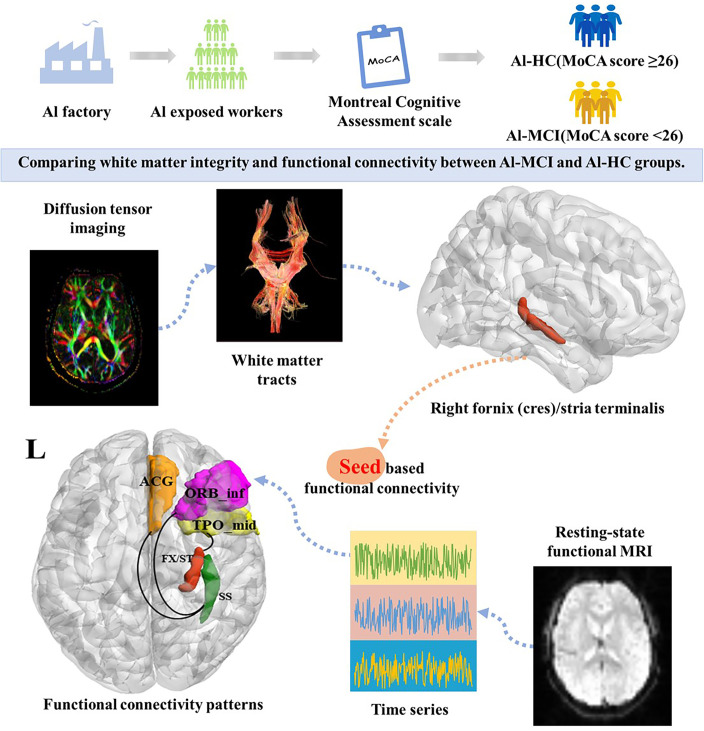
Visual Abstract

## Significance Statement

Our study illuminates the neural “pathway” linking long-term aluminum (Al) exposure to mild cognitive impairment. Through integrated plasma Al assessments, cognitive evaluations, and advanced neuroimaging, we unveil differences in white matter integrity and functional connectivity, particularly in the right fornix/stria terminalis. These findings elucidate the neurobiological mechanisms underlying Al-induced MCI, highlighting the importance of addressing occupational Al exposure as a modifiable risk factor for cognitive decline.

## Introduction

Aluminum (Al) is the third-most abundant elements in Earth's crust, following oxygen and silicon. It is widely applied in various fields such as food, cosmetics, and pharmaceutical production and is commonly encountered in the general population (Kandimalla et al., 2016). In comparison, workers in Al smelting, welding, and other manufacturing industries may experience higher levels of exposure (Klotz et al., 2017). One concerning aspect of Al exposure is its ability to cross the blood–brain barrier (BBB) and accumulate in the brain over extended periods, leading to neurotoxic effects (Yokel, 2006). Prolonged exposure to Al has been linked to neurological dysfunction, characterized by decreased learning and memory abilities, as well as cognitive impairment (Giorgianni et al., 2014). A longitudinal study has also shown the impact of long-term occupational Al exposure on cognitive function (Lu et al., 2021). Therefore, the first aim of this study was to explore the relationship between plasma Al concentration and cognitive impairment.

Mild cognitive impairment (MCI) is a transitional state between normal aging and dementia (Petersen et al., 1999). The changes of white matter (WM) integrity can be seen in MCI. A meta-analysis has revealed that MCI is associated with widespread microstructural alterations in WM throughout the brain (Sexton et al., 2011). Compared with normal aging adults, individuals with MCI showed abnormal WM tracts in various regions, including the fornix (cres)/stria terminalis (FX/ST) and sagittal stratum (SS; J. Liu et al., 2013; Zhuang et al., 2013; Shim et al., 2017; Yu et al., 2017). Additionally, other related studies have found structural degradation of the hippocampus-temporal and thalamus-related fibers in MCI individuals, and they are significantly correlated with cognitive scores (Zhou et al., 2022). One of the analytical techniques employed with resting-state functional magnetic resonance imaging (rs-fMRI) data is functional connectivity (FC), which has shown promising results for the diagnosis of MCI (Ibrahim et al., 2021; Y. Zhang et al., 2022). Differences in FC between individuals with MCI and healthy control (HC) were mainly observed in the default mode network (DMN), dorsal attention network, and frontoparietal task network, with the DMN exhibiting the most important discrimination ability (X. Xu et al., 2020). Additionally, other studies have reported reduced FC in the hippocampus, medial prefrontal cortex, and middle temporal gyrus in the sensorimotor network (Cai et al., 2017). Apart from the DMN-related regions, one study identified abnormal FC in the basal ganglia of MCI individuals, which are closely related to cognitive scores. And it also highlights that the amygdala is critical in the early detection of MCI (Xiong et al., 2022). Overall, WM and FC alterations are present in individuals with MCI and may be potential neural mechanism of MCI caused by Al exposure.

Interestingly, previous research has revealed that the blood oxygenation level-dependent (BOLD) signal in WM can also reflect the neural activity in the brain (Ding et al., 2016). And Peer et al. (2017) reported that the functional network constructed by WM is not only related to the gray matter (GM) functional network but also to the structure of WM. Numerous studies have illustrated alterations in WM functional networks among individuals with schizophrenia (Y. Jiang et al., 2019, 2022; Fan et al., 2020). Since the most previous studies on MCI have constructed FC networks between GM regions, considering that WM tightly connects different GM regions and occupies nearly half of the human brain (Wang et al., 2022), the present study will further explore the role of WM FC in Al-induced MCI. Therefore, in this study, WM with structural changes obtained by diffusion tensor imaging (DTI) analysis was utilized as a seed to construct a seed-based FC network. Meanwhile, both structural information from DTI and functional information from rs-fMRI were combined to comprehensively explore the neurophysiological mechanism of MCI induced by Al exposure.

The aim of this study is to investigate the differences in WM integrity and FC between participants with Al-induced MCI and normal participants and to explore the neural mechanisms underlying Al-induced MCI. Firstly, correlation analysis was employed to examine the relationship between plasma Al concentration and cognitive function scores. Based on previous results, we predicted that the higher the plasma Al concentration, the worse the cognitive function (Giorgianni et al., 2014; Lu et al., 2021; F. Zhang et al., 2023). Subsequently, tract-based spatial statistics (TBSS; Smith et al., 2006) was utilized to investigate the changes of WM microstructure between individuals with Al-induced MCI and HC. We hypothesize that the WM fiber bundles related with memory cognition may display structural changes in these individuals (Sexton et al., 2011; J. Liu et al., 2013; Zhuang et al., 2013; Shim et al., 2017; Yu et al., 2017; Zhou et al., 2022), and furthermore, we hypothesize that these WM fiber bundles may exhibit significant BOLD signals. Finally, FC was constructed using the altered WM tracts as seeds to explore the correspondence between these WM regions and GM or other WM tracts throughout the brain in MCI. We propose that in individuals with Al-induced MCI, there may be abnormalities in the FC of WM regions closely linked to cognitive functions.

## Materials and Methods

### Participants

A total of 52 male workers exposed to occupational Al >10 years, who were examined in the First Hospital of Shanxi Medical University from October 2014 to November 2019, were enrolled in this study. The participants were divided into two groups according to their Montreal Cognitive Assessment (MoCA) score: the healthy control group (Al-HC, MoCA score ≥26) and MCI group (Al-MCI, MoCA score <26). The study was approved by the Ethics and Human Committees of Shanxi Medical University, and all participants signed an informed consent form. The exclusion criteria were (1) systemic or neuropsychiatric disorders, (2) family history of neurodegenerative diseases, (3) history of medications that affect cognition, (4) abnormal infarction or focal lesion, (5) inability to complete neuropsychological assessment, (6) contraindications to MRI, and (7) left-handed people.

### Plasma Al concentration determination

Fasting cubital venous blood was collected from the participants in the morning, and 2 ml whole blood was collected through a heparin sodium anticoagulated tube. The plasma was separated by centrifugation at 1,000 rpm within 10 min. Subsequently, 0.4 ml of plasma was thoroughly mixed with 1.6 ml nitric acid (4%) and left at room temperature for 24 h. And then the samples were analyzed using inductively coupled plasma mass spectrometry to measure the concentration of Al in the plasma. Each sample was determined twice, and the average of the two measurements was recorded as the final result.

### Neurocognitive assessments

The cognitive function of the participants was evaluated using the MoCA scale (Nasreddine et al., 2005; Jia et al., 2021), which is a widely used tool for screening and assessing cognitive impairment. A MoCA score of <26 indicated cognitive impairment.

### MRI data acquisition

MRI examinations were performed for all participants using a 3.0 T MRI scanner (Skyra, Siemens) with a 32-channel array coil. The following images were acquired: The DTI data were acquired using an echo planar imaging (EPI) sequence with the following setting: time repetitive (TR)/time echo (TE), 9,100/84 ms; flip angle, 90°; field of view (FOV), 112 × 112 mm^2^; matrix size, 112 × 112; voxel size, 2 × 2 × 2 mm^3^; slice thickness, 2 mm; number of slices, 75; and *b* = 1,000 s/mm^2^. To ensure image quality during the acquisition process, we used 64 diffusion-weighted directions for encoding and conducted water phantom calibration before acquiring data for each batch of participants to ensure data quality and accuracy. The rs-fMRI data were acquired using an EPI sequence with the following parameters: TR/TE, 2,000/30 ms; flip angle 90°; FOV, 1,344 × 1,344 mm^2^; matrix size, 64 × 64; voxel size, 3.5 × 3.5 × 3.5 mm^3^; slice thickness, 3.5 mm; 33 slices and 240 volumes; and acquisition time, 8 min. During the scanning, all participants were required to keep their eyes closed, remain awake, and avoid considering anything. The high-resolution T1–weighted images were acquired using a magnetization-prepared rapid gradient-echo sequence. The sequence parameters were as follows: TR/TE, 2,530/2.01 ms; flip angle, 7°; FOV, 256 × 256 mm^2^; matrix size, 256 × 256; slice thickness, 1 mm; and 192 slices.

### DTI data preprocessing and TBSS analysis

The DTI data were preprocessed using FMRIB Software Library v6.0 (FSL, https://fsl.fmrib.ox.ac.uk/fsl, version 6.0). For each participant, the DTI data underwent correction for eddy current distortions and head movement using the Eddy Current Correction tool in FSL. Nonbrain tissues were then removed using the Brain Extraction Tool from FSL. Finally, diffusion index maps including fractional anisotropy (FA), mean diffusivity (MD), radial diffusivity (RD), and axial diffusivity (AD) were calculated using the FSL diffusion tensor analysis toolkit.

Next, statistical analysis of the FA, MD, AD, and RD images was performed using the TBSS pipeline (Smith et al., 2006). The FA images of all participants were aligned into the standard space (FMRIB5, 1.0 × 1.0 × 1.0 mm^3^ MNI 152 space) using FMRIB's nonlinear image registration tool. Then a mean FA skeleton was created, and all individual participants’ aligned FA were projected onto the mean FA skeleton. The same steps were applied to individual AD, RD, and MD images. Then, the JHU DTI-based WM atlas was used to identify the WM structural characteristics of different fiber bundles (Mori et al., 2008).

### Functional data preprocessing and seed-based FC analysis

The MRI data preprocessing was carried out with the SPM 12 (www.fil.ion.ucl.ac.uk/spm) and MATLAB 2013b (www.mathworks.com) software. The high-resolution T1–weighted MRI data underwent segmentation to GM, WM, and cerebrospinal fluid (CSF) using SPM 12. For the rs-fMRI data, the following primary processing steps were performed. Firstly, we discarded the first 10 volumes to reduce scan noise and magnetic field instability and corrected slice timing to align the slices temporally. Secondly, rs-fMRI data with excessive head motion (translation > 2.5 mm or head rotation > 2.5°) were removed. Thirdly, we registered the high-resolution T1–weighted images and corrected rs-fMRI images to align the structural and functional data. Next, linear trends removal and bandpass filtering (0.01–0.1 Hz) were performed. Finally, to investigate the functional roles of GM and WM, we removed CSF signals and 24 head motion parameters while preserving the signals from WM and GM.

Seed-based FC analysis was performed in MATLAB 2013b. WM regions showing significant differences in FA between Al-MCI and Al-HC were selected as seed regions. An automated anatomical labeling (Tzourio-Mazoyer et al., 2002) atlas was applied to segment the GM into 90 regions, and the JHU DTI-based WM atlas was applied to identify the WM. The average time series of BOLD signals for each ROI were extracted and Pearson's correlational coefficients were used to establish seed-based FC maps. Fisher's *r* to *z*-transform was analyzed to improve normality.

### Statistical analyses

All demographic and neuropsychological statistical analyses were performed using the Statistical Product and Service Solutions software (Version 22.0). Categorical variables were reported as counts, while continuous variables were reported as mean ± standard error (*x̄ *± SE). Normal distribution of continuous variables was assessed using the two independent samples of *t* test, and nonnormally distributed variables were analyzed using the Mann–Whitney *U* test. A significance level of *p *< 0.05 was considered statistically significant.

Firstly, the relationship between plasma Al concentrations and MoCA scores was explored with gender, age, education level, income, and expose time as covariates. Correlations were considered significant at two-tailed *p *< 0.05. Then, to investigate the differences in FA values and FC changes between Al-MCI and Al-HC groups, a two-sample *t* test was employed. Due to multiple comparisons, false discovery rate (FDR) correction was applied to adjust *p* values (*p *< 0.05).

### Data and code availability

The data that support the findings of this study are available from the corresponding author upon reasonable request.

## Results

### Demographic and clinical characteristics

The demographic and clinical characteristics of all participants are shown in Table 1. A total of 52 individuals under Al exposure participated in our study. Based on the MoCA score, 28 participants exhibited MCI, while the remaining 24 participants showed no evidence of cognitive impairment. There were no significant differences between the Al-MCI and Al-HC groups in terms of age (*t *= −0.209; *ν *= 50; *p *= 0.835), income (*t *= 1.564; *ν *= 50; *p *= 0.124), expose time (*t *= −1.794; *ν *= 50; *p *= 0.079), and plasma Al concentration (*t *= 1.612; *ν *= 50; *p *= 0.113). However, compared with the Al-HC group, the Al-MCI group exhibited inferior performances on education level (*t *= −3.296; *ν *= 50; *p *= 0.002) and MoCA score (*t *= −15.217; *ν *= 50; *p *< 0.001). Furthermore, controlling for age, gender, education level, income, and exposure time, a significant correlation was observed between plasma Al concentration and MoCA score (*p *= 0.028; *r *= −0.304).

**Table 1.** Demographic and clinical characteristics of Al-MCI and Al-HC groups

	Al-MCI	Al-HC	SE	95%CI (lower)	95%CI (upper)	*t*	*p* value
Gender (male/female)	28/0	24/0					
Age (years)	47.32 ± 1.07	47.63 ± 0.95	1.450	−3.215	2.608	−0.209	0.835
Education level (years)	9.43 ± 0.37	11.25 ± 0.41	0.553	−2.931	−0.711	−3.296	0.002*
Income	3.00 ± 0.05	2.83 ± 0.10	0.107	−0.047	0.381	1.564	0.124
Expose time (years)	24.25 ± 1.81	28.17 ± 1.05	2.183	−8.302	0.469	−1.794	0.079
Plasma Al (μg/L)	50.63 ± 9.26	31.77 ± 6.54	11.704	−4.644	42.373	1.612	0.113
MoCA score	18.68 ± 0.46	27.67 ± 0.34	0.591	−10.174	−7.802	−15.217	<0.001*

Al, aluminum; MCI, mild cognitive impairment; HC, health control; MoCA, Montreal cognitive assessment.

* Significant differences were found between Al-HC and Al-MCI groups.

### Difference in WM integrity between the Al-HC and Al-MCI groups

**Figure 1.**
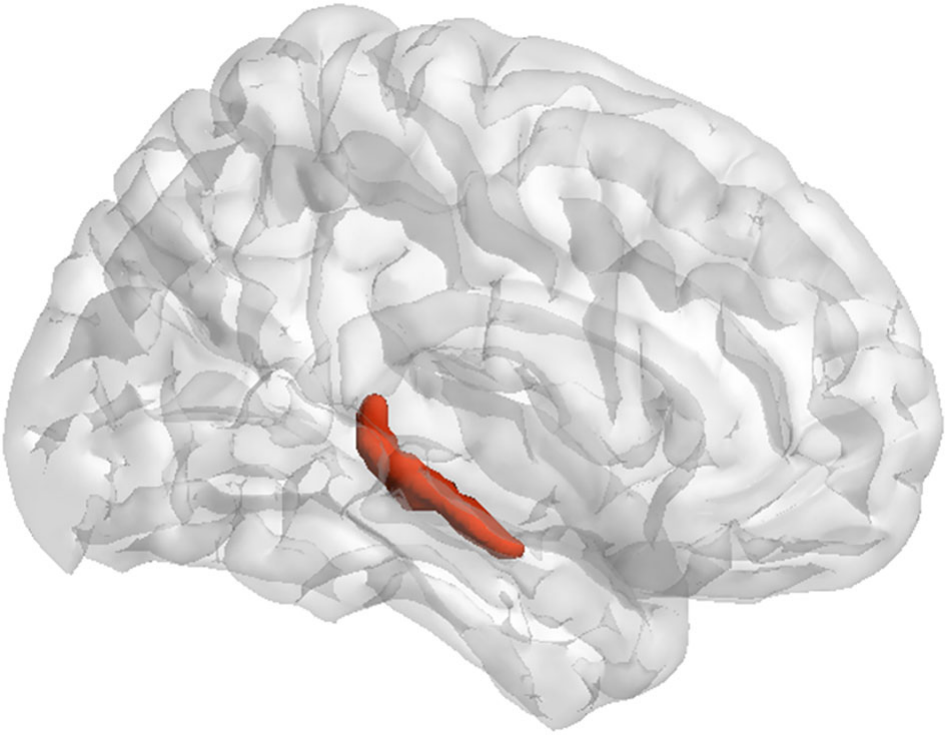
Localization of the right FX/ST region. Results from the whole-brain TBSS analysis revealed that only the FA values of the right FX/ST exhibit statistically significant differences.

The TBSS analysis revealed significant differences in WM integrity between the Al-HC and Al-MCI groups. Specifically, the FA values for the right FX/ST were significantly higher in the Al-MCI group (*t *= 2.809; *ν *= 50; *p *= 0.007) compared with those in the Al-HC group. Conversely, the MD and RD values for the right FX/ST were reduced in the Al-MCI group (MD, *t *= −2.165; *ν *= 50; *p *= 0.035; RD, *t *= −3.121; *ν *= 50; *p *= 0.003). Although the lower FA value of the left FX/ST can be observed in the Al-MCI group compared with that in the Al-HC group, the difference between the two groups did not attain statistical significance. The detailed results are listed in Table 2, and Figure 1 shows the location of the right FX/ST.

**Table 2.** Neuroimage findings between Al-MCI and Al-HC groups

	Al-MCI	Al-HC	SE	95%CI (lower)	95%CI (upper)	*t*	*p* value
TBSS analysis							
FX/ST. L							
FA (×10^−5^)	55,094.53 ± 540.03	55,728.30 ± 562.63	782.070	−22,046.050	937.063	−0.810	0.422
FX/ST. R							
FA (×10^−5^)	55,214.16 ± 434.61	53,231.60 ± 569.74	705.905	564.712	3,400.417	2.809	0.007*
MD (×10^−5^)	76.13 ± 0.51	77.80 ± 0.58	0.768	−3.207	−0.120	−2.165	0.035*
AD (×10^−5^)	127.98 ± 0.84	127.83 ± 1.15	1.400	−2.662	2.960	0.107	0.915
RD (×10^−5^)	50.23 ± 0.54	52.75 ± 0.61	0.810	−4.157	−0.901	−3.121	0.003*
Seed-based FC analysis							
ORB_inf. R - SS. R	0.48 ± 0.04	0.60 ± 0.04	0.050	−0.224	−0.022	−2.461	0.018*
ACG. R - SS. R	0.47 ± 0.03	0.61 ± 0.03	0.047	−0.235	−0.044	−2.948	0.005*
SS. R - FX/ST. R	0.68 ± 0.02	0.76 ± 0.02	0.030	−0.132	−0.012	−2.412	0.020*
FX/ST. R - TPO_mid. R	0.18 ± 0.04	0.30 ± 0.04	0.054	−0.228	−0.010	−2.206	0.033*

Al, aluminum; MCI, mild cognitive impairment; HC, health control; FX/ST, fornix (cres)/stria terminalis; L, left; FA, fractional anisotropy; R, right; MD, mean diffusivity; AD, axial diffusivity; RD, radial diffusivity; ORB_inf, inferior frontal gyrus, orbital part; SS, sagittal stratum; ACG, anterior cingulate and paracingulate gyri; TPO_mid, temporal pole, middle temporal gyrus.

* Significant differences were found between Al-HC and Al-MCI groups after FDR correction.

### Difference in FC of the right FX/ST between the Al-HC and Al-MCI groups

**Figure 2.**
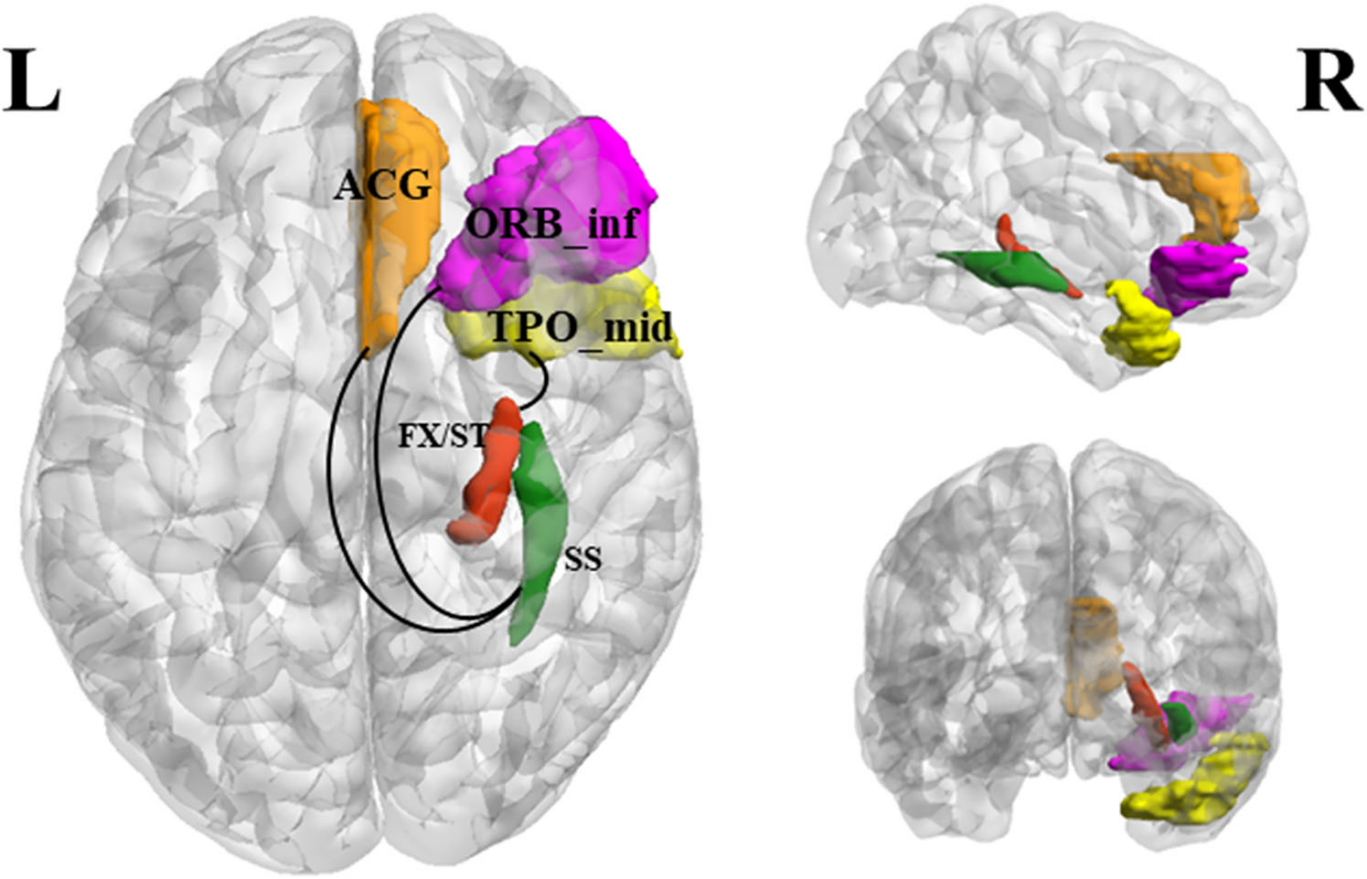
FC patterns associated with the right FX/ST. Seed-based FC analysis was performed using the right FX/ST as the seed region. In the Al-MCI group, reduced FC was observed between the right FX/ST and right TPO_mid, as well as SS. Additionally, decreased FC was noted between the right SS and right ACG and ORB_inf. These findings suggest the establishment of a “pathway” involving these WM fibers and cortical areas. Specifically, the connectivity from the right temporal pole to the right FX/ST, then to the right SS, and further to the right anterior cingulate and paracingulate gyri and right inferior frontal gyrus, orbital part. L, left; R, right; ACG, anterior cingulate and paracingulate gyri; ORB_inf, inferior frontal gyrus, orbital part; TPO_mid, temporal pole, middle temporal gyrus; FX/ST, fornix (cres)/stria terminalis; SS, sagittal stratum.

A total of five subjects were excluded from the rs-fMRI analysis due to excessive head movement during the scan. Consequently, the final analysis included 25 with cognitive impairment and 22 participants without cognitive impairment. To investigate the FC patterns associated with the right FX/ST, seed-based FC was analyzed. Compared with the Al-HC group, the Al-MCI group exhibited significantly decrease FC between the right FX/ST and the right temporal pole: middle temporal gyrus (TPO_mid; *t *= −2.206; *ν *= 45; *p *= 0.033) and right SS (*t *= −2.412; *ν *= 45; *p *= 0.020). Furthermore, reduced FC was also observed between the right SS and right anterior cingulate and paracingulate gyri (ACG; *t *= −2.9489; *ν *= 45; *p *= 0.005) as well as the right inferior frontal gyrus, orbital part (ORB_inf; *t *= −2.461; *ν *= 45; *p *= 0.018; Table 2). A “pathway” was formed by these WM fibers and cortical areas. Specifically, the “pathway” defined as connectivity from the TPO_mid to the FX/ST, then to the SS, and further to the ACG and ORB_inf showed differences between Al-MCI group and Al-HC group (Fig. 2).

## Discussion

Chemical metal elements, such as iron and Al, play crucial roles in human health and disease. Humans are exposed to these metals through ingestion, inhalation, and skin contact (Igbokwe et al., 2019). Iron is essential and must be maintained at optimal levels; deficiencies or excesses can impair function. In contrast, nonessential metals like Al can cause dysfunction and toxicity even at low concentrations (Huat et al., 2019). Both Al and iron can cross the BBB (Kandimalla et al., 2016) and induce neurotoxicity through increased oxidative stress, protein modifications, and inflammation. Al competes with iron for binding sites, increases intracellular free iron, and promotes reactive oxygen species production while reducing antioxidant enzyme activity, exacerbating neuronal damage (Fattoretti et al., 2003; Sánchez-Iglesias et al., 2009). Both metals enhance tau protein phosphorylation, leading to neurofibrillary tangles (Huat et al., 2019), and may increase amyloid-beta production and aggregation (Ricchelli et al., 2005; Drago et al., 2008; Boopathi and Kolandaivel, 2016), contributing to Alzheimer's disease. Heavy metal exposure also activates microglia and astrocytes, elevating inflammatory cytokines, which modulate tau phosphorylation (Quintanilla et al., 2004; C. Jiang et al., 2018).

In this study, DTI and rs-fMRI were employed to investigate the structural and functional differences in individuals with and without MCI exposed to Al. After controlling for age, education, income, and working years, a negative correlation was revealed between plasma Al concentration and MoCA scores. The FA of right FX/ST increased in MCI participants, while MD and RD of FX/ST were decreased. The seed-based FC analysis revealed that the connectivity between the right FX/ST and the right SS and TPO_mid was decreased, as well as the connectivity between the right SS and the right ACG and ORB_inf. These significant findings may be potential neural mechanism of Al-induced MCI.

### Relationship between plasma Al concentration and MoCA score

Previous research has consistently reported a strong association between Al and cognitive impairment (Flaten, 2001). Our study further supports these findings by demonstrating that long-term Al exposure can lead to cognitive dysfunction. Consistent with previous studies (Lu et al., 2021; S. M. Xu et al., 2022), we observed a significant negative correlation between plasma Al concentration and MoCA scores. Specifically, as plasma Al concentration increased, MoCA scores decreased, indicating the neurotoxic effects of prolonged Al exposure.

### Alterations in WM integrity of the right FX/ST in participants with Al-MCI

In our study, we observed significant differences in WM integrity between the Al-HC and Al-MCI groups, specifically localized to the right FX/ST region. The FX/ST is a neural pathway that connects various regions, with the fornix comprising axons from the hippocampus and the stria terminalis comprising axons from the amygdala. These regions are connected with the hypothalamus and the limbic system (Wakana et al., 2004). The FA differences in our study were confined to the right FX/ST, possibly because it is composed of small fibers and plays a crucial role in cognitive function, making it susceptible to Al exposure, which leads to specific microstructural changes in WM. Previous studies have also found a strong association between the FX/ST and clinical impairment in MCI individuals (Zavaliangos-Petropulu et al., 2019; Srisaikaew et al., 2020), and changes in FA values of the fornix have been effective in distinguishing between HC and MCI participants (Bozoki et al., 2012). However, contrary to our findings, these studies reported reduced FA values in the fornix and stria terminalis, indicating a decrease in WM integrity (Y. Liu et al., 2011; Bozoki et al., 2012; Zavaliangos-Petropulu et al., 2019).

In our study, the increased FA value of the right FX/ST may be related to myelin content (Alves et al., 2015), while the significantly decreased RD value without differences in AD indicates a breakdown of myelin integrity without axonal structural damage (Di Paola et al., 2010; Alves et al., 2015). This indicates that WM damage in participants with Al-induced MCI may involve myelin breakdown followed by repair, leading to increased myelin thickness and more directional water diffusion. We also observed that the FA value of the left FX/ST was lower in the Al-MCI group compared with that in the Al-HC group, but the difference did not reach statistical significance. This is not sufficient to support that the increased FA value in the right FX/ST is a compensation for the decreased FA value in the left FX/ST. Similar to the increased FA observed in the motor pathways of Parkinson's disease patients (Mole et al., 2016), we believe the FA increase in Al-MCI participants in our study could result from an adaptive response to prolonged Al exposure, reflecting neuroplasticity of neural circuits. Additionally, Al exposure might activate glial cells, increasing local inflammatory cytokines and triggering a moderate neuroinflammatory response that helps repair and stabilize WM fibers (Ekdahl et al., 2009). These factors could all contribute to the increased FA value in the right FX/ST.

### Alterations of FC in participants with Al-MCI

Previous research has shown that the BOLD signal in WM appears to reflect intrinsic neural activity (Ding et al., 2018). In this study, we found a significant reduction in FC between the right FX/ST and the right SS in participants with MCI compared with that in Al-HC. This reduction suggests the existence of neural activity in SS, which could be a crucial factor in the decline of cognitive function among individuals with MCI under the Al exposure. Previous studies have regarded SS as an important WM fiber of MCI (Yu et al., 2017). SS is a primary corticocortical subcortical WM fiber, including inferior longitudinal fasciculus (ILF) and inferior fronto-occipital fasciculus (IFOF). The IFOF connects the occipital cortex, temporal basal area, superior parietal lobule, and anterior cuneus to the frontal lobe (Martino et al., 2011), playing a vital role in language function (Panesar et al., 2017). The ILF is directly connects the occipital cortex and temporal lobe and is connected to the anterior hook bundle to transmit information to the orbitofrontal part of the brain (Ashtari, 2012; Bajada et al., 2017). It carries sensory, visual, auditory, and somatosensory information from the posterior to the anterior brain regions. Previous studies have demonstrated that the impairment of the ILF or IFOF was associated with memory, attention, and executive dysfunction in people with MCI (Cho et al., 2008; Hodgetts et al., 2017; Chen et al., 2020). Based on the decline in FC between the right FX/ST and SS in our study, we speculate that it could provide valuable insights into the potential cognitive deficits in MCI individuals under Al exposure.

In addition, we also found reduced FC in right TPO_mid, ORB_inf, and ACG in the Al-MCI group. The TPO_mid plays a critical role in social cognition and semantic processing (J. Xu et al., 2019). And the ACG is essential components of the limbic system, responsible for regulating both cognitive and emotional processing (Bush et al., 2000; Mohanty et al., 2007). The inferior frontal gyrus, including the ORB_inf, is related to various cognitive functions, including attention, motor inhibition and execution, reasoning, and social cognition (Hartwigsen et al., 2019). All three GM regions belong to the DMN (Buckner and DiNicola, 2019), an important brain network involved in higher-order cognition processing (Smallwood et al., 2021). Consistent with our results, a meta-analysis showed that ACG could serve as a potential imaging marker for MCI (Song et al., 2021). Numerous studies have reported disruptions in the DMN in individuals with MCI (Y. Li et al., 2016; Xue et al., 2019; X. Li et al., 2020). Thus, we propose that the abnormal FC between the right FX/ST and SS with DMN regions in this study may be related to the cognitive changes in individuals with Al-MCI, which could be helpful to identify MCI participants under the Al exposure.

There are some limitations to this study: (1) The sample size is relatively small, and the study includes only male subjects. To enhance the robustness of our findings, future research should expand the sample size and include a more diverse population. (2) This study is a retrospective study. In the future, we will conduct a longitudinal study investigating the relationship between cognitive deterioration and brain structure and function. (3) In this study, both MCI and HC subjects were exposed to Al environment. To better understand the impact of Al exposure on cognitive impairment, future investigations should include a control group consisting of individuals without occupational Al exposure. Additionally, recruiting Alzheimer's disease patients exposed to Al environments could offer information about the developmental mechanisms of cognitive impairment.

## Conclusion

Our study demonstrated changes in the structural and FC of WM and GM regions in participants with Al-induced MCI, suggesting that the “pathway” formed among these regions may play a significant role in Al-induced MCI. It provides new insights into the neural mechanism of Al-induced MCI.

## Synthesis

Reviewing Editor: Niraj Desai, National Institute of Neurological Disorders and Stroke Decisions are customarily a result of the Reviewing Editor and the peer reviewers coming together and discussing their recommendations until a consensus is reached. When revisions are invited, a fact-based synthesis statement explaining their decision and outlining what is needed to prepare a revision will be listed below. The following reviewer(s) agreed to reveal their identity: NONE.

Thank you for making such substantial revisions, which have strengthened the manuscript and the interpretation considerably.

One last reviewer point for you to consider regarding the Visual Abstract: "It does not currently include information about the two groups and the main group comparison being made."
